# The origin and evolution of shrews (Soricidae, Mammalia)

**DOI:** 10.1098/rspb.2024.1856

**Published:** 2024-12-18

**Authors:** Haobo Yuan, Ephriam D. Dickson, Quentin Martinez, Patrick Arnold, Robert J. Asher

**Affiliations:** ^1^ Department of Zoology, University of Cambridge CB2 3EJ, UK; ^2^ Department of Biosystems Science and Engineering, ETH Zürich, Switzerland; ^3^ Carlsbad Caverns National Park, USA; ^4^ Staatliches Museum für Naturkunde Stuttgart, Stuttgart DE-70191, Germany; ^5^ Evolutionary Adaptive Genomics, Institute of Biochemistry and Biology, University of Potsdam, Germany

**Keywords:** timetree, sesamoid, dental pigmentation, mosaic evolution, soricinae, crocidurinae

## Abstract

Shrews are among the most speciose of mammalian clades, but their evolutionary history is poorly understood. Their fossil record is fragmentary and even the anatomy of living groups is not well documented. Here, we incorporate the oldest, most complete fossil shrew yet known into the first phylogenetic analysis of the group to include molecular, morphological and temporal data. Our study reveals previously unknown diversity among total- and crown-group soricids. This includes a novel element of the mammalian skeleton: a robust, needle-like sesamoid extending cranially from the second thoracic neural arch in Myosoricini, comparable in length to these species' humeri. Additionally, ‘red-toothed’ shrews have an unusually elongate basicranium, and ‘white-toothed’ shrews probably evolved from a common ancestor with dental pigmentation. The fossil *Domnina* and crown soricids have a double-jaw articulation and incomplete zygomatic arch, but unlike nearly all crown species, *Domnina* has open vomeronasal canals and a tympanic process of the basisphenoid. *Domnina* and other heterosoricids are phylogenetically outside crown Soricidae. The oldest, well-supported total-group soricoids are North American, not Asian, and Soricidae probably originated during the Palaeocene or early Eocene. The diverse mammalian genus *Crocidura* originated and began to diversify during the Miocene.

## 1. Background

Shrews (Soricidae, Lipotyphla) have been characterized as ‘among the most ancient of all living mammals’ with ‘unspecialized body plans, almost unchanged since they evolved about 45 million years ago’ [1]. Yet the group possesses some of the most novel traits among mammals, such as a medially pocketed coronoid process without a masseteric fossa on its lateral surface and an uncalcified deciduous dentition [2]. Perhaps their most unusual feature is the double jaw joint [3], in which two distinct, bony articular surfaces on the squamosal articulate with corresponding facets on the mandibular condyle [4]. Fossils of the oldest, widely accepted fossil shrew, *Domnina*, show distinct facets on the mandibular condyle that are closer together than those of extant species [5,6], but the anatomy of the squamosal side of the jaw articulation in any Palaeogene soricid has been unknown, until now. Some features of modern soricids have only recently been described, such as the position of the jaw joints lateral, rather than posterolateral, to the nasal fossa [7] and differences in the size and shape of the middle ear roof and ectotympanic rings [8]. The proximal humerus of soricids has pectoral and deltoid processes and a teres tubercle [9]. Most living species exhibit a broad distal styloid process of the ulna [10] and fusion of the scaphoid and lunate carpal bones [11].

The fossil record of soricids consists primarily of isolated jaws and teeth of Eocene and younger ages in Eurasia and North America [4,12,13]. Well-preserved, cranial remains are rare and most associated specimens are Miocene or younger [14–16]. The oldest, undisputed total-group genus, *Domnina*, is known from isolated teeth from the middle Eocene [17,18], fragmentary jaws [4,13,19,20] and a partial rostrum with an ear region [5] from late Eocene through Oligocene deposits in North America. Fossils of *Soricolestes* from the middle Eocene of Mongolia comprise several lower jaw fragments, which Lopatin [21] interpreted as more closely related to soricids than other extant mammalian groups (but see [22]).

Here, we describe the oldest skull and jaws of a fossil shrew associated with postcrania: University of Wyoming (UW) 57130, an early Oligocene specimen of *Domnina gradata* from the Brule Member of the White River Formation of southeastern Wyoming, USA. We phylogenetically analyse this specimen along with 22 other, pre-Holocene fossils and 44 recent species of lipotyphlans, focusing on the Soricidae, based on a morphological-molecular matrix consisting of 217 cranioskeletal characters, plus six nuclear and 15 mitochondrial genes. We apply molecular and stratigraphic data combined with a Bayesian morphological clock [23] to estimate the temporal pattern behind their evolution. Using artificial extinction [24], we also quantify the extent to which our sample of morphological characters accurately reconstructs the phylogeny of recent taxa with well-corroborated affinities. With these data, we seek to establish the branching structure and chronology of soricid evolution and to uncover previously undocumented skeletal features of shrews.

## 2. Methods

### (a) Morphological data

We coded 217 morphological characters for 44 living (electronic supplementary material, appendix S1) and 23 fossil terminals (electronic supplementary material, appendix S2). 74 characters were from the non-dental cranium, 89 from the jaws and dentition and 54 from the postcranial skeleton. We used terminals at species level, except *Scapanus*, *Erinaceus*, *Limnoecus*, *Nesophontes*, *Oligosorex* and *Srinitium*, for which we coded multiple species per genus to reduce missing data (electronic supplementary material, appendices S1 and S2). We relied primarily on micro-computed tomography (CT) scans to code morphology and rendered three-dimensional volumes with Drishti 4.0 [25,26]. Graphic illustrations of our character states are available via our morphobank project page (electronic supplementary material, appendix S3).

**Figure 1.**
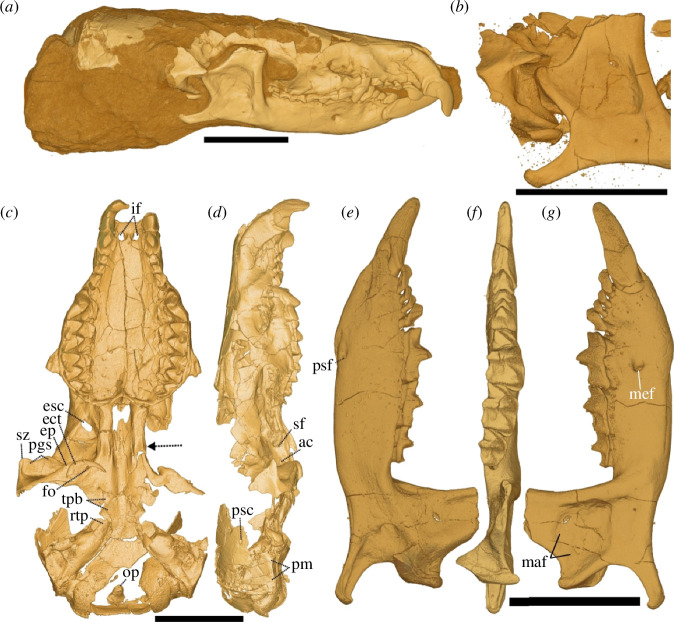
*Domnina gradata* UW 57130 skull *in-situ* (*a*) and close-up of jaw articulation (*b*); virtually prepared skull in ventral (*c*) and lateral (*d*) views; lower right jaw in medial (*e*), occlusal (*f*), and lateral (*g*) views. ac = alisphenoid canal, ect = ectotympanic, ep = entoglenoid process, esc = ethmoid-sinus canal foramen (enlarged postmortem), fo = foramen ovale, if = incisive foramina, op = odontoid process of C2, pgs = superior glenoid process, pm = petromastoid, psc = posterior sinus canal foramen, rtp = rostral tympanic process of petrosal, sf = sphenorbital fissure, sz = squamosal root of zygoma, tpb = tympanic process of basisphenoid. Dashed arrow in (*c*) shows approximate posterior extent of the nasal fossa. Scale bars = 5 mm.

**Figure 2.**
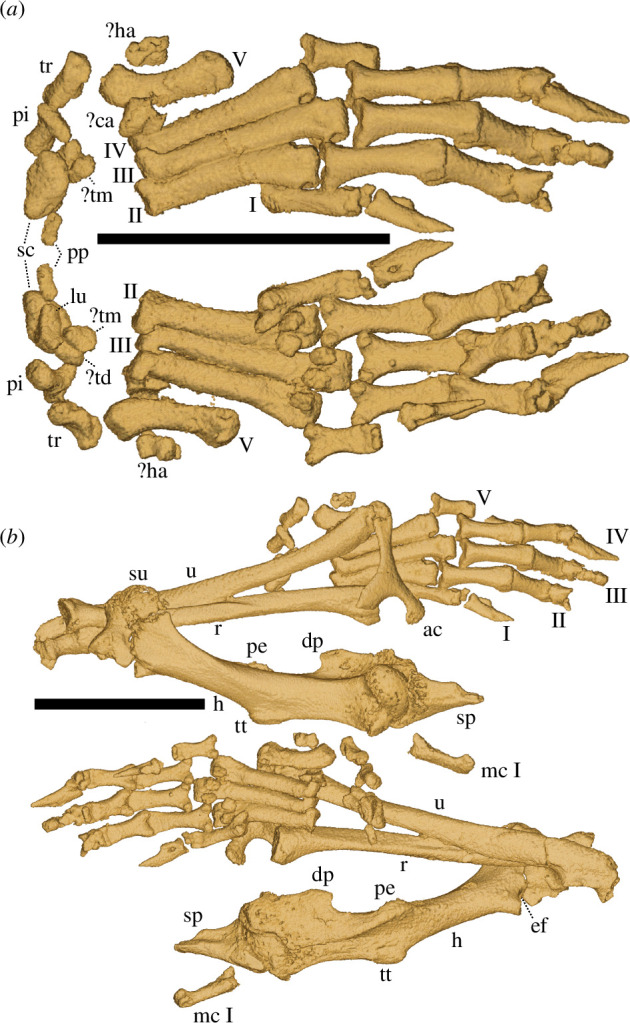
Left manus (*a*) and forelimb (*b*) of *D. gradata* UW 57130 in (from top) dorsal and ventral views. ac = scapular acromion, ca = capitate, dp = deltoid process, ef = entepicondylar foramen, h = humerus, ha = hamate, lu = lunate, mc = metacarpal, pe = pectoral process, pi = pisiform, pp = prepollux, r = radius, sc = scaphoid, sp = scapula, su = supinator crest, td = trapezoid, tm = trapezium, tr =triquetrum, tt = teres tubercle, u = ulna, Roman numerals I–V = digits. Scale bars = 5 mm.

Our specimen of *D. gradata* (UW 57130) consists of a skull, lower jaws (figure 1), articulated left forelimb (figure 2), and distal right humerus from the Brule Member of the White River Formation in Converse County, near Douglas WY, and dates to the earliest Oligocene (Orellan). Locality and stratigraphic data are on file at the UW, Laramie. The collection area is between two dated ash beds, near the base of the Brule, and has yielded a diverse fauna [27,28]. UW 57130 may be from the same sedimentary horizon as USNM 12841, a *D. gradata* specimen figured by McDowell [5], collected ‘7 miles S.E. Douglas Wyo’ by G.F. Sternberg in 1931.

Simpson [20] regarded the molar structure of *D. gradata* as ‘practically identical’ with that of *Domnina thompsoni*, named as a new species based on its smaller size and distinct dental formula. Repenning [4] figured both species and noted that, as in UW 57130, *D. gradata* has four, single-rooted, lower antemolars between the enlarged incisor and the first molar, vs. five in *D. thompsoni*. Molars of UW 57130 fit into the ranges reported by Simpson [20] for *D. gradata* and are considerably larger than those of *D. thompsoni*. We therefore recognize UW 57130 as a specimen of *D. gradata*.

### (b) DNA alignment

We sampled 15 mitochondrial and six nuclear genes from Genbank (electronic supplementary material, appendix S4), forming an 18762 bp alignment from 28 soricids, four erinaceids, nine talpids, two solenodontids and the recently extinct *Nesophontes*. We sought taxa at least 50% complete for the majority of the 43 living taxa in our sample but added less complete taxa in order to sample key branches of the lipotyphlan radiation (electronic supplementary material, table S1). We added 26 insertion/deletion (indel) characters. Molecular data were missing for all fossils except *Nesophontes* [29]. We designed a quality-control approach to filter out potentially artefactual GenBank sequences (electronic supplementary material, table S2). For each locus, we employed the ‘FindLongestSeq’ function from AnnotationBustR to identify the longest sequence for each species from the search results. We then applied the neighbour-joining method in PAUP* 4.0a [30] to analyse all sequences retrieved in each search to infer a distance-based phylogeny. We examined the placements of the longest sequences identified by ‘FindLongestSeq’ on the neighbour-joining tree, adhering to the criteria defined in the electronic supplementary material, table S2. Two RAG1 sequences did not meet these criteria: JN414936 (originally *Solenodon paradoxus*) and LC124967 (originally *Notiosorex crawfordi*). The BLAST search for JN414936 returned best hits for *Equus quagga* and *Pongo abelii*; LC124967 returned *Homo sapiens*. We therefore replaced JN414936 with AY530075 for *Solenodon paradoxus* and left RAG1 for *Notiosorex crawfordi* as missing. Where possible, we used complete mitochondrial genomes and sequences curated by NCBI staff.

We aligned recent taxa (including *Nesophontes*) using Clustal Omega [31] and AliView [32], optimizing manually using Muscle [33]. We removed leading and trailing sequences to ensure the remaining sites were at least 20% complete and checked that alignments began at a first codon position and ended at a third. We minimized the occurrence of stop codons; when present, we put them towards alignment ends. We transformed contiguous, triplet gaps within the alignments of APOB, BDNF, BRCA1, GHR, ND4, ND5 and ND6 into 26, non-autapomorphic, binary characters. For non-coding ribosomal RNA, we excluded nucleotide blocks with ambiguous base homology. The electronic supplementary material, appendix S5 contains our concatenated alignment and morphological data in nexus format.

### (c) Models of sequence evolution

We applied IQ-Tree 2.2.2.6 [34] to select optimal partitioning schemes and models of sequence evolution. We converted the combined FASTA alignment into PHYLIP format using ‘convertFasta2Phylip.sh’ [35] and specified ‘-m MFP+MERGE’ to select for the best-fit partitioning scheme and models, similar to PartitionFinder [36] but with a FreeRate model [37]. We allowed IQ-Tree to determine the optimal number of cores using ‘-nt AUTO’. Using a Bayesian information criterion, IQ-Tree identified 18 partitions that we used for maximum likelihood (ML) analyses (electronic supplementary material, table S3).

Using the partitioning scheme and models shown in the electronic supplementary material, table S3, we estimated two initial ML phylogenies with IQ-Tree 2.2.2.6, one including all 67 taxa and the other 44 recent taxa known for molecular data, and used the latter as a starting tree for our non-clock Bayesian analysis. For morphology, we used the Mk model [38] and ascertainment bias correction (‘ASC’) and identified 21 ordered characters with the Mk model allowing exchange of neighbouring states only and ‘ASC’. We could not employ ‘ASC’ for unordered characters in the recent-taxon ML analysis in IQ-Tree owing to the presence of three constant sites. We modelled gap characters as a single partition with the general time reversible model (‘GTR2’) and ‘ASC’ (electronic supplementary material, table S3). We set search parameters in IQ-Tree to default values. We used the ‘GENESITE’ method to resample partitions and then sites within resampled partitions [39,40] and performed 1000 ultrafast bootstrap replicates [41] to generate a consensus tree, forming a starting topology for non-clock Bayesian analyses.

We also used IQ-Tree to determine optimal partitioning schemes (electronic supplementary material, table S3) in our Bayesian analyses, as above, and specified ‘-m TESTMERGEONLY’ and ‘-mset mrbayes’ to select for the best-fit scheme and test only nucleotide models available in MrBayes. We placed gap characters into a single restriction site partition and used the F81-like binary model [42]. We placed the morphological characters into a single morphology partition, of which 21 characters were ordered and 187 unordered, with the default Mk model. For both indel and morphology partitions, we used the ‘lset variable’ option for ascertainment bias and modelled gamma-shaped rate heterogeneity. We unlinked state frequencies, substitution rates, gamma-shape parameters, proportion of invariable sites and transition/transversion rate ratios across all partitions. We ran four independent runs and eight chains each in parallel on 32 cores, discarded the first 25% as burn-in and established convergence based on average standard deviation of split frequencies near or below 0.01, effective sample size using Tracer 1.7.3 [43] of at least 200 and usually well over 1000, and potential scale reduction factor at or near 1.0. For Bayesian analyses of extant taxa, we ran at least 8 million generations, and for fossils at least 15 million, sampling every 2000. We used *Solenodon-Nesophontes* to root topologies [29].

### (d) Timetree analysis

For our analyses incorporating stratigraphic data, we explored narrow and broad fossil priors to account for uncertainty regarding fossil ages. We defined narrow uniform priors following the upper and lower marine stage boundaries that correspond with each fossil’s earliest occurrence, with the upper boundary tied to the youngest age of the marine stage. We assigned broad uniform priors by extending the lower boundary. This was tied to the bottom of a previous marine stage in which ample deposits are known where the fossil could have occurred, but is nonetheless absent [44]. In both cases, the fossils' actual tip age in each posterior topology are informed by the morphological clock [23], with a stronger assumption about the lower boundary using narrow priors.

We ran both approaches with different combinations of tree age priors and topological constraints. In the first combination, we set the tree age prior to the lower boundary of the oldest fossil (*Soricolestes*) using a truncated normal distribution (offset = 72.3, mean = 72.4, s.d. = 5 with broad priors; offset = 47.8, mean = 47.9, s.d.= 5 with narrow priors) and no topological constraint. In the second combination, the same tree age prior was combined with the relationships among extant lipotyphlan families constrained to the topology resulting from the non-clock Bayesian analysis, as shown in the electronic supplementary material, figure S1a and equivalent to (Solenodontidae, (Erinaceidae, (Talpidae, Soricidae))). Because *Soricolestes* may be too nested to reliably constrain the lipotyphlan root, we used stratigraphic occurrence data from *Adunator ladae* from the Torrejonian of North America [44] to inform the tree age prior in a further analysis. We used an offset gamma distribution to model a hard minimum boundary at 61.6 Ma (million years ago), peak probability at 67 to 73 Ma and a soft maximum boundary at 160 Ma [44,45].

Alignments, partitions and models of sequence evolution were the same as in the non-clock analyses. We derived a clock rate prior from path lengths of the non-clock MrBayes tree, and the mean age of the fossil tips using an R script from [46]. This suggested a gamma distribution (shape parameter = 2.1, rate parameter = 115.52) as a starting point for the clock rate. We used the independent gamma rates model to estimate relaxed clock rate variation, and a fossilized birth-death prior on branch lengths with a speciation prior (‘speciationpr’) of exp(10) and flat priors for extinction (‘extinctionpr’) and fossilization (‘fossilizationpr’) priors. We set the sampling probability of terminal lineages (‘sampleprob’) to 0.09, as our sample of 44 extant species comprises 9% of extant lipotyphlan species. Using either 32 or 56 cores per node on our cluster, we ran (respectively) four Markov chain Monte Carlo runs with eight chains each, or eight runs with seven chains, for at least 15 million generations, sampling every 2000 generations and discarding the first 25% as burn-in. We summarized trees with ‘sumt contype = allcompat’ and determined convergence as in the non-clock Bayesian analyses, above. Posterior probabilities in our timetree are equivalent to the majority-rule proportions among the sampled, post-burn-in topologies.

### (e) Artificial extinction

Artificial extinction [24] tests the assumption that fossilizable data yield accurate results. If true, we would expect some degree of statistical consistency as more such data are added to a given phylogenetic problem. Felsenstein [47] made this point in reference to statistical estimation methods, not particular types of data, but the principle is the same. In our case, we ask if congruence with an independent, well-corroborated tree increases with ever increasing samples of fossilizable data.

To address this issue, we treated living species with well-supported phylogenetic affinities as though they were fossils, deleting non-fossilizable data and retaining only those anatomical characters present in a fossil. In our matrix, we sampled 44 taxa with molecular data and 23 fossils without; the former were ‘subjects’ and the latter ‘templates’. We explored every subject (excluding the root)-template combination, or 43×23 (= 989) individual phylogenetic analyses of recent taxa, treating each subject as if it were a fossil, retaining only anatomical characters present in the respective template and using the character states of the subject. For the 217 morphological characters in our matrix, templates ranged in completeness from 11% (*Soricolestes*) to 69% (*D. gradata*; see the electronic supplementary material, table S1).

In addition to the first set of 43 subjects artificially fossilized using 23 templates, we undertook further analyses in which multiple subjects were fossilized per analysis, all using the same template, first with the subjects' real states and again with random binary states. We randomly picked 2, 4, 8, 16 or 32 subjects to be artificially fossilized. Altogether, this yielded 11 868 distinct phylogenetic analyses: 989 for each set of 1, 2, 4, 8, 16 or 32 artificially fossilized subjects using real states ( = 5934), and a further 5934 analyses with subjects coded using randomized binary states. We used implied-weights parsimony [48,49] in TNT [50] as our optimality criterion with a concavity value of 1. Taking only a few seconds or minutes per analysis, this enabled the nearly 12 000 individual phylogenetic analyses to be completed within a reasonable time frame.

We measured congruence of a strict consensus from each analysis with our topology of recent taxa based on our DNA alignment plus insertion-deletion characters, collapsing nodes with posterior probabilities under 0.8 and nodes that conflicted with recent, genomic phylogenies [51]. We refer to this as our well-corroborated tree (electronic supplementary material, figure S2) and measured congruence with this tree using two indices of congruence: shared splits and shared quartets [24]. Our expectation is that as the amount of fossilizable data increases, congruence with the independently derived, well-corroborated tree will also increase.

## 3. Results and discussion

### (a) Novel element of the mammalian skeleton

**Figure 3.**
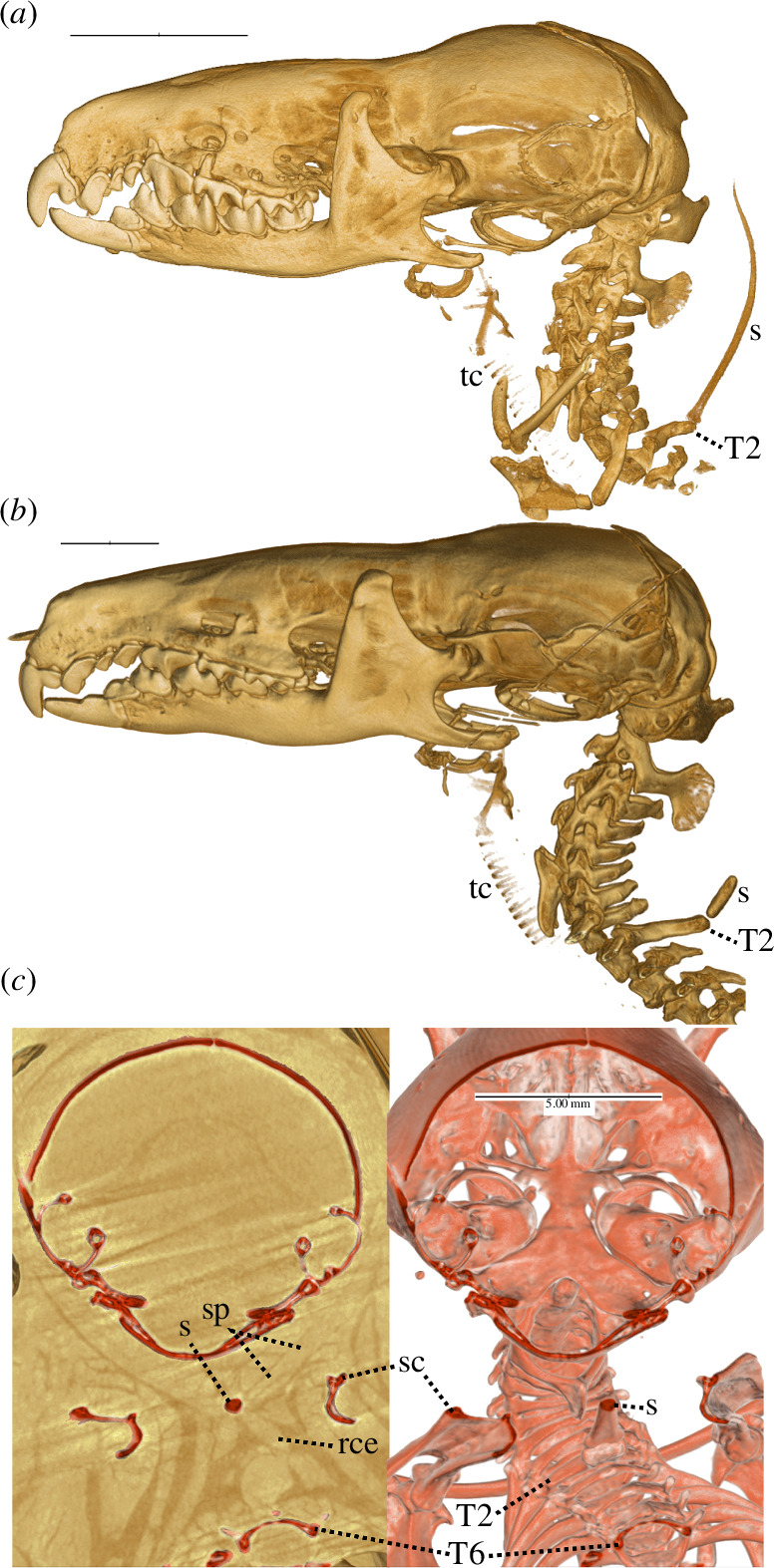
Lateral (*a, b*) and postero-dorsal (*c*) views of the head and neck region in alcohol-preserved, intact specimens of *Congosorex verheyeni* (*a*, SMNS 50411), *Surdisorex norae* (*b*, FMNH 190260) and *Congosorex phillipsorum* (*c*, FMNH 177721). Posterodorsal view (*c*) is of the same semi-transverse section, osteology in red, with (left) and without (right) reconstructed soft-tissues evident in CT scan. rce = rhomboideus cervicis, s = nuchal sesamoid, sc = scapula, sp = splenius, tc = tracheal cartilages, T2 = second thoracic vertebra, T6 = sixth thoracic vertebra. Note greater opacity in nuchal sesamoids compared to tracheal cartilages. Oblique streaks in (*c*, left) and through the hyoid, angular process of dentary, ectotympanics and posterior skull in (*b*) are CT artefacts. Scale bars = 5 mm.

Among the most surprising results of our analysis is the discovery of a robust sesamoid in Myosoricini (see taxonomy in the electronic supplementary material, appendix S6), denser than nearby tracheal cartilages, similar in opacity to adjacent vertebral bone, and extending dorso-cranially from the second thoracic neural arch (figure 3; electronic supplementary material, table S4) towards the nuchal region. In *Myosorex* and *Congosorex* (electronic supplementary material, appendix S7), the nuchal sesamoid is elongate and comparable in proximo-distal length to these species' humeri. It is shorter in the fossorial *Surdisorex* but still robust (figure 3*b*). Lin *et al*. [52] noticed a structurally similar but smaller sesamoid in species of *Crocidura*, *Suncus* and *Scutisorex*, observations which we confirm in our sample (electronic supplementary material, figure S3, table S4). Lin *et al*. [52] noted the element’s role in anchoring the splenius muscle and discussed how it may increase neck mobility. Our anatomical observations are consistent with their interpretation, although we cannot rule out additional functions, such as sensation or defence, particularly given the extraordinary length of the sesamoid in *Congosorex* and *Myosorex*. In *Congosorex phillipsorum*, the three most conspicuous muscles attaching to the nuchal sesamoid are splenius, rhomboideus capitis and rhomboideus cervicis (figure 3*c*), as shown in our animated CT volume (electronic supplementary material, appendix S7). Splenius originates along the proximal half of the sesamoid and inserts on the lateral surface of the anterior petromastoid (or ‘tabular’ of Sharma [53]). Both rhomboid muscles insert on the posterior end of the vertebral border of the scapula; rhomboideus capitis originates from the middle region of the sesamoid and rhomboideus cervicis originates from its cranial region (electronic supplementary material, appendix S7).

We do not yet know the function of the robust nuchal sesamoid in Myosoricini. In specimens of *Myosorex* for which we have sex identifications (electronic supplementary material, table S4), the sesamoid : humerus length ratio is approximately 0.9 and broadly overlaps among males and females, suggesting that it is not the result of sexual selection. The sesamoid is embedded within soft tissues (electronic supplementary material, appendix S7) and, despite its needle-like appearance (figure 3), does not appear to protrude beyond the skin. There is no obvious lumen or groove along which glandular secretions could have been transported. Again, this is consistent with, but not exclusive to, a function related to mobility [52].

The nuchal sesamoid would be unlikely to survive skeletonization techniques typically applied in most museums, and is evident only among intact, alcohol-preserved or cleared-and-stained specimens. This is probably why it has remained undocumented in the published literature until now. For example, Sharma [53] correctly noted in *Suncus murinus* that ‘the spinous process of the second thoracic vertebra’ (to which the sesamoid attaches) ‘is well developed’, but mentioned sesamoids only in reference to the manus and pes. *Crocidura russula* (electronic supplementary material, figure S3) and all other species of Crocidurinei observed to date (electronic supplementary material, table S4) nonetheless show a smaller version of the element seen in Myosoricini, as does the erinaceid *Hylomys suillus* (electronic supplementary material, figure S3a). Several talpids exhibit an elongate, midline element dorsal to cervical vertebrae and caudal to the skull. This approaches T2 in some specimens of *Uropsilus* (electronic supplementary material, figure S3b), but the sesamoid in talpids lacks a clear bony connection to their more gracile T2 neural arch.

### (b) Mosaic evolution of shrews

*Domnina* and most other heterosoricids show eight or nine teeth in each dental quadrant, rather than six in most crown soricids, and have a relatively deep masseteric fossa without a medially pocketed coronoid. Our new specimen (UW 57130) shows that *Domnina* also has an alisphenoid canal (a conduit anterior to the foramen ovale that opens into the sphenorbital fissure) and a tympanic process of the basisphenoid that contributes to at least a partial auditory bulla (figure 1). Maier *et al*. [7] noted that the jaw articulation of extant soricids is anteriorly situated and laterally brackets the ethmoidal recess of the nasal fossa. By contrast, the jaw articulation of *Domnina* is posterolateral to the nasal fossa (figure 1), as in non-soricids. Furthermore, the ethmoid foramina of *Domnina* are positioned anterior to the sphenorbital fissure, and its posterior sinus canal foramina are positioned anterior to its petromastoid, rather than anterodorsal (figure 1). In each of these regards, *Domnina* departs from modern shrews and resembles non-soricids such as *Uropsilus*, *Hylomys* and *Solenodon*.

The UW *Domnina* skull is the only Palaeogene soricoid that, to our knowledge, exhibits an intact glenoid fossa of the squamosal, which shows two widely spaced articular facets (figure 1; electronic supplementary material, figure S4). The corresponding mandibular facets, while dual, are situated immediately adjacent to each other, as opposed to those in crown soricids, which are more widely separated [4,6]. McDowell [5] went so far as to claim that the mandibular condyle of *Domnina* ‘is nearly identical, except in size, with that of *Solenodon*.’ *Domnina* and *Solenodon* do share a medially extensive condyle, but that of *Domnina* is more obliquely oriented with the lower articular facet offset ventrally from the upper (electronic supplementary material, figure S4). The squamosal articular surfaces for the condyle in *Domnina* are extensive both ventrally and dorsally; they comprise distinct surfaces separated by a non- or minimally articular region, thereby resembling the condition seen in soricids such as *Crocidura olivieri*, not *Solenodon* (electronic supplementary material, figure S4). We interpret the morphology of *Domnina*, with widely spaced squamosal but closely spaced mandibular facets, as representative of an ancestral state from which the more derived conditions evolved.

In addition to the double jaw articulation, soricids have an unusually elongate basicranium with an unfused roof of the middle ear, known as the median lacerate foramen [8] (alternatively ‘piriform fenestra’ of [5]). This gap anterior to the bony housing of the cochlea is not unique to soricids, but is particularly large in this group (electronic supplementary material, figure S5). Maier *et al*. [7] suggested that the forward movement of the soricid jaw articulation contributed to the elongate basicranial region, and, along with the double-jaw joint, facilitated a tweezer-like function for the elongated, anterior incisors. Additionally, Maier *et al*. [8] noted the relatively smaller ectotympanic ring, and longer median lacerate foramen, in soricines (i.e. species of Soricinae) compared to those of crocidurines (i.e. species of Crocidurinae, see the electronic supplementary material, appendix S6).

Our results place these observations in a phylogenetic context and highlight just how different soricines are, not only compared to other lipotyphlans, but also to other shrews, perhaps as a result of their distinctive brain structure [54]. The basicranium as a proportion of overall cranial length (electronic supplementary material, figure S5) in soricines ranges from 39–47%. On average, soricines show 43%, compared to 39% among crocidurines, 34% among talpids, 27% in erinaceids and 23% in *Solenodon*. With its basicranium comprising 37% of overall cranial length, *Domnina* falls below the mean of crocidurines and represents an intermediate value between soricines and other lipotyphlan groups. Optimized onto our phylogeny, lipotyphlans exhibit a gradual increase in the proportion of the skull occupied by the basicranium towards the soricine branches of the phylogeny (electronic supplementary material, figure S5).

Other poorly understood features of *Domnina* include its zygomatic arch and nasal fossa. Repenning [4] argued that *Domnina* retained a complete zygomatic arch, whereas McDowell [5] claimed the opposite. We support McDowell’s view; UW 57130 has a reasonably intact, right squamosal root of the zygoma which lacks an anterior extension (figure 1). Its maxillary root is slightly abraded and is larger than that generally present in soricids. Furthermore, the nasal fossa in *Domnina* and non-soricids opens into the oral cavity via paired incisive foramina, as opposed to the single, median palatine foramen exhibited by most crown soricids (electronic supplementary material, figure S6). *Domnina* and most other non-soricids exhibit grooves on either side of the anterior nasal septum that receive the paired vomeronasal organ, located along the anterior floor of the nasal fossa, without a complete bony covering laterally or dorsally. By contrast, the bony depressions for the vomeronasal organs in crown soricids are enclosed anteriorly by bone, forming channels at the anterior ends of the vomeronasal organ. Additionally, the infraorbital canal in *Domnina* is long, exceeding in length its mediolateral breadth, similar to those of Soricinae and Myosoricini, but shorter than the infraorbital canal of Crocidurinei. *Domnina* exhibits a slightly enlarged infraorbital foramen, above the average among ambulatory species but relatively smaller than that of semiaquatic soricids [55].

Our specimen of *Domnina* (UW 57130) is the only heterosoricid with a three-dimensionally preserved cranium associated with articulated postcranial elements (figure 2). These comprise a left forelimb with a distal scapula, humerus, radius-ulna, carpals and manus. Most carpal elements are intact, including the terminal phalanges and even the minute sesamoids ventral to the distal ends of the metacarpals. The Miocene taxon *Lusorex* is also known from postcrania [15], but the specimen is flat and many anatomical details are therefore obscure. The forelimb of *Domnina*, in contrast, is well preserved, and shows a mosaic of features seen in extant groups. The scapula resembles those of Myosoricini and Soricinae in lacking a supraspinatous fossa that extends to the glenoid facet for the humerus. Like most non-*Sorex* soricines, *Domnina* shows unfused scaphoid and lunate bones of the manus (figure 2). Crocidurines differ by exhibiting scapho-lunate fusion, and species of Crocidurinei possess a supraspinatous fossa that extends laterally to its glenoid articulation for the humerus. In other regards, the shoulder joint of *Domnina* resembles that of crocidurines, with a pectoral process of the humerus situated distal to the teres tubercle (figure 2), not opposite as in most soricines. *Domnina* furthermore has a prepollux, like crocidurines, but unlike soricines such as *Cryptotis* [11] and *Sorex*.

### (c) Dental pigmentation

Pigmentation of soricid teeth has long informed the dichotomy between ‘red-toothed’ (soricine) and ‘white-toothed’ (crocidurine) shrews, although these categories lack a consistent anatomical relationship with enamel microstructure [56]. Pigmentation results from the sequestration of iron oxides into dental tissues [57], leading to red coloration along functionally relevant parts of the toothrow, such as the anterior band of enamel on rodent incisors or on cusp tips in most soricines. Past authors have inferred pigmentation in fossils, including *Domnina*, based on direct observation and ultraviolet light; pigmentation blocks the fluorescence typically emitted by mammalian enamel [19]. By contrast, UW 57130 lacks marked colour variation on the sides vs. tips of any dental locus. Its enamel fluoresces weakly in response to ultraviolet light, but the matrix in which some of the fossil is still encased does so even more (electronic supplementary material, figure S7). Voyta *et al*. [57] showed that ultraviolet light does not consistently distinguish between taphonomic and biological causes of tissue fluorescence. Pending further analysis, we are not certain if the apparent lack of pigmentation in UW 57130 is real or an artefact of post-depositional taphonomy.

However, several heterosoricids have been interpreted to show pigmentation, including other specimens of *Domnina* [56]. Pigmentation has been reported in at least some species of the Oligocene fossils *Srinitium* [58], *Oligosorex* [59] and the Miocene *Shargainosorex* [16], here placed on the crocidurine stem. The phylogenetic distribution of this character suggests that the common ancestor of total-group Soricoidea exhibited dental pigmentation, which was then secondarily lost among crocidurines, comparable to the evolution of pigment-free soricines such as *Anourosorex* [57].

### (d) Timing of the shrew radiation

**Figure 4.**
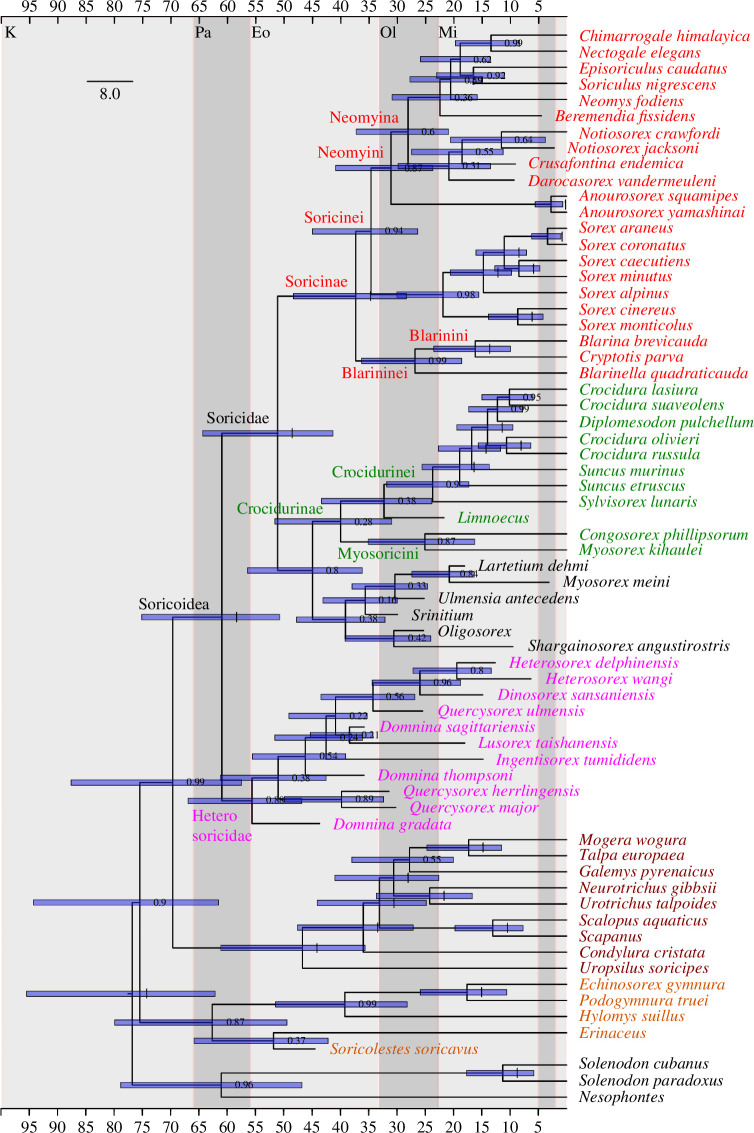
Optimal Bayesian timetree derived from 15 million generations, burn-in 25%, narrow priors, extant taxa constrained using the optimal, non-timetree, Bayesian topology: (*Solenodon*,(*Erinaceus*,(*Galemys*,*Blarina*))). Root node calibrated with *Adunator* following [44]. Horizontal blue bars represent 95% credibility intervals of divergence time estimates. Numbers adjacent to nodes show posterior probabilities. Grey taxa are solenodontoids, light brown erinaceids, brown talpids, pink heterosoricids, green crocidurines, red soricines. Geological ages are Cretaceous (K), Palaeocene (Pa), Eocene (Eo), Oligocene (Ol) and Miocene (Mi), corresponding to the *x*-axis scale.

Our optimal timetree (figure 4) accounts for all of the anatomical, molecular and stratigraphic information available from our dataset, and is therefore the basis for our conclusions. We provide details on our non-timetree analyses in Methods, and on taxonomy in the electronic supplementary material, appendix S6. Age estimates for the oldest, most basal nodes in our phylogeny are the least precise, with the widest 95% credibility intervals (or HPD for 'highest posterior density' following [60]). More nested nodes show increasingly narrower bounds (figure 4) using either narrow or broad temporal priors (electronic supplementary material, appendix S8). Furthermore, overlap among credibility intervals derived from narrow and broad priors increases with distance from the root.

Based on our timetree with narrow priors (figure 4), our estimated, median age for the origin of total group Soricoidea is in the Palaeocene, with a wide credibility interval between 75−51 Ma. The initial split within crown soricids probably took place during the Eocene, with a 95% credibility range between 64−41 Ma. These ranges are congruent with estimates from Brace *et al*. [29] and the reanalysis by Springer *et al*. [61] of data from Sato *et al*. [62]. While an ancient, Mesozoic origin for one or more high-level lipotyphlan groups is possible [61], none of our credibility intervals is exclusively pre-Palaeogene. With the important qualification that divergence estimates (particularly for basal nodes) are imprecise, we estimate that the diversification of Soricinae began during the late Eocene, possibly as old as 48 and as recently as 28 Ma. The crown crocidurine radiation is largely Eurasian and African and would have begun to diversify between 52 and 31 Ma. We agree with Repenning [4] that the North American *Limnoecus* is more closely related to crocidurines than soricines. Furthermore, we hypothesize that this genus, with a first occurrence in the early Miocene of Nebraska, USA [63], should have a lineage extending well into the Oligocene or even Eocene. With some qualifications (discussed below), our analysis weakly supports the middle Miocene *Shargainosorex* as a stem crocidurine. *Shargainosorex,* along with taxa previously classified as ‘crocidosoricines’ [64,65] including *Ulmensia*, *Srinitium*, *Oligosorex, Myosorex* and *Lartetium*, appear together but are not well-supported on the crocidurine stem in our timetree (figure 4). Extant *Myosorex* appears in a clade with the fossil *Myosorex meini* only in our implied-weighted parsimony topology (electronic supplementary material, figure S8B); neither parsimony nor Bayesian non-clock (electronic supplementary material, figure S8A) analyses support ‘crocidosoricine’ monophyly.

With nearly 200 species, *Crocidura* is a very diverse mammalian genus [66]. Our sample of this clade (four species of *Crocidura* and *'Diplomesodon' pulchellum*) is minute, but still provides a divergence estimate consistent with those of Flynn *et al*. [67] and Furió *et al*. [68]. The former authors identified isolated teeth of *Crocidura* sp*.* from the Chinji Formation of Pakistan, approximately 14 Ma in age. With a 95% credibility interval from 23−12 Ma using narrow priors (or 27−12 Ma with broad), and using calibrations (electronic supplementary material, appendix S8) other than that proposed for *Crocidura* by Flynn *et al*. [67], our estimate for the last common ancestor of *Suncus murinus* and *Crocidura* brackets their palaeontological estimate. All of the divergence estimates for this and other soricid nodes from Brace *et al*. [29] are within our credibility intervals (electronic supplementary material, appendix S9). By contrast, other molecular clock estimates [69–71] are younger. For example, Dubey *et al*. [69] hypothesized 8 Ma for the common ancestor of *Crocidura*, and 9.3 Ma for the common ancestor of *Crocidura* with a paraphyletic *Suncus*, substantially younger than the estimate of Flynn *et al*. [67]. For *Crocidura*, we estimate an origin between 19−10 Ma. The highest-level clade with a divergence estimate figured by Dubey *et al*. [69] was 16.5 Ma for Crocidurinae, substantially postdating our estimate between 31 and 52 Ma (figure 4; electronic supplementary material, appendix S9).

### (e) Anatomical predictions among fossils

The diversity of crown soricids, mapped onto our optimal phylogeny, enables us to make predictions about as-yet unknown anatomical regions in fossil taxa, such as the mid-Eocene *Soricolestes* and the enigmatic Oligocene-Pliocene ‘Crocidosoricinae’. The nuchal sesamoid is one such character. Given the presence of at least a small sesamoid in all crocidurines we have observed to date, we would expect such an element to have been present in at least some fossils on the stem to Crocidurinae. We cannot say exactly where on the crocidurine stem the nuchal sesamoid evolved, but we regard it as more likely to have been present among one or more crown crocidurines such as *Limnoecus*, and stem crocidurines such as *Oligosorex*, *Srinitium* and *Ulmensia,* than among any fossil soricines. While sesamoids are rarely preserved in the fossil record, presence of a nuchal sesamoid may be inferred by the elongate, robust spinous process of the second thoracic vertebra with which it articulates. This structure is more gracile among soricines (electronic supplementary material, figure S3D) than crocidurines (electronic supplementary material, figure S3C).

Crocidurines are the only lipotyphlans in our dataset to exhibit alisphenoid fenestrae (electronic supplementary material, figures S5, S6). This gap in the basicranium opens into the superior orbital fissure, or the intracranial space housing cranial nerves V−1 and V−2 and leading to the sphenorbital fissure [7]. Crocidurines in our dataset except *Suncus murinus* exhibit a superior orbital fissure positioned far enough ventrally so as to cause this gap in the basicranium, medial and slightly posterior to the foramen ovale. The alisphenoid fenestra is sometimes confused with the vidian foramen or foramen ovale, but there are crocidurine specimens that show all three structures (electronic supplementary material, figure S6). If our Bayesian topologies (figure 4; electronic supplementary material, figure S8a) are correct, and once the relevant anatomical regions are found, we would expect one or more stem crocidurines, such as *Srinitium* and *Oligosorex*, and fossils within the crocidurine crown, such as *Limnoecus*, to exhibit alisphenoid fenestrae.

An unexpected result of our Bayesian topologies was the reconstruction of the Eocene taxon *Soricolestes* [21] closer to erinaceids than to soricids. In the timetree (figure 4), *Soricolestes* appeared within Erinaceidae with weak support. In the unconstrained Bayesian analysis excluding stratigraphic data (electronic supplementary material, figure S8a), *Soricolestes* appeared more strongly supported among erinaceids, but was unresolved within that clade. Our optimal parsimony topology (electronic supplementary material, figure S8b) placed *Soricolestes* in a polytomy at the base of Soricoidea, consistent with the interpretation of Lopatin [21].

*Soricolestes* is the least complete fossil (missing 89% of the 217 morphological characters) in our sample and retains scant anatomical evidence with which to have confidence in either placement. Its dental anatomy resembles that of soricids in some regards [21], but there are similarities to non-soricids as well, such as the prominent talonid cusp on its last premolar [21]. If *Soricolestes* really is more closely related to erinaceids than soricids, we would expect future discoveries of more complete fossils to exhibit a limited range of character states. Total-group erinaceids might show, for example, a foramen ovale positioned anteromedial to its entoglenoid process, dual ectopterygoids, a patent and relatively narrow alisphenoid canal with an anterior opening lateral to the sphenorbital fissure, a subarcuate fossa that dorsally contacts the sidewall of the braincase, a prominent mastoid tubercle, a reduced caudal tympanic process of the petrosal, a ventrally closed tubal canal, a single articulation for the lower jaw on the squamosal, or a patent postglenoid foramen. Graphic illustrations of these and other character states are available on our morphobank.org project page (electronic supplementary material, appendix S3).

### (f) Phylogenetic value of fossilizable data

Uncertainty regarding the phylogenetic affinities of fossils such as *Soricolestes* and *Shargainosorex*, or even anatomically better-known taxa such as *D. gradata*, underscores an important fact: fossils retain a tiny fraction of the data available from living species to undertake phylogeny reconstruction. It is therefore worth asking if such small packets of fossilizable data retain phylogenetic signal. Using artificial extinction [24], we determined that congruence with an independent, well-corroborated tree (electronic supplementary material, figure S2) increases with the addition of fossilizable data (electronic supplementary material, figure S9). In other words, the more hard-tissue characters are added to taxa for which all DNA characters are missing, the higher the resulting congruence with an independent, well-corroborated phylogeny will be. This is true for analyses artificially fossilizing one or many taxa at a time; indeed, the slope of improving congruence with fossil completeness increases when multiple subjects are fossilized, although overall similarity to the well-corroborated tree decreases as more subjects are treated as fossils (electronic supplementary material, figure S9). We therefore conclude that morphological characters available in fossils contribute positively to accurate phylogeny reconstruction.

This is obviously not a guarantee that any single estimate for a given fossil is correct. We expect that, compared to well-known fossils, incomplete ones such as *Soricolestes*, known for just 11% of our morphological characters, will be more subject to error and more likely to undergo changed phylogenetic interpretations with future discoveries. As shown in the electronic supplementary material, figure S9, artificial extinction experiments that sampled the 26 of 217 morphological characters in *Soricolestes* accurately reconstructed an average of 89% of extant subject taxa treating just one subject as an artificial fossil, dropping to an average of 52% treating eight subjects simultaneously as artificial fossils, and 18% treating 32 subjects as artificial fossils. This compares to values of 94%, 76% and 48% for the equivalent analyses using *D. gradata* as a template, our most complete fossil known for 149 of 217 morphological characters, and now the anatomically best-known Palaeogene shrew.

Our analysis confirms that, along with other heterosoricids, *Domnina* is the sister taxon to living shrews, as inferred by previous investigators based on far less anatomical information [4,19,20]. This is itself a demonstration of the information content of fossilizable data. Moreover, the anatomy of living and fossil soricoids shows that, far from being ‘almost unchanged’ [1], the radiation of modern shrews has undergone substantial mosaic evolution since they diverged at least 55 Ma from the common ancestor they shared with other lipotyphlan mammals, and since they diversified at least 40 Ma into the very speciose clade we know today.

## Data Availability

All of our data are available via the electronic supplementary material [72] and appendices. Our morphological database (appendix S3) is available at http://morphobank.org/permalink/?P4835. Our animation of Congosorex anatomy (appendix S7) is available at https://youtu.be/yhBURNBefGI. Appendix S4 provides Genbank accession numbers.
